# RNF114 Interacts with EWSR1 to Regulate VEGFR2 in HER2-positive Breast Cancer

**DOI:** 10.7150/jca.106001

**Published:** 2025-02-28

**Authors:** Yingchuan Zhu, Yue Song, Yilu Lu, Wenhao Jiang, Jingfei Zhang, Lan Yin, Xinyu Lin, Dachang Tao, Yongxin Ma

**Affiliations:** Department of Medical Genetics, Frontiers Science Center for Disease-related Molecular Networks, State Key Laboratory of Biotherapy, West China Hospital, Sichuan University, Chengdu, China.

**Keywords:** RNF114, HER2-positive breast cancer, VEGFR2, EWSR1, proliferation, autophagy

## Abstract

RNF114, a member of the E3 ubiquitin ligase family, was first identified as a zinc-binding protein that exhibits frequent genomic amplification across various cancers. Previous studies have shown that inhibition of RNF114 E3 ligase activity by Nimbolide treatment can result in trapping of PARP1 and synthetic lethality in BRCA-mutated cancers, suggesting its E3 ligase role in tumor progress. However, it's important to reveal novel functions and interacting molecules of RNF114. Here, we first described that RNF114 promotes tumor proliferation and autophagy by interacting with EWSR1 and regulating VEGFR2 expression in HER2-positive breast cancer (BC). Our results also showed that RNF114 is significantly overexpressed in BC and is associated with TNM stage and poor prognosis in BC patients. And knockdown of RNF114 suppresses proliferation, migration, invasion, and autophagy of HER2-positive BC cells. Our findings highlight the transcriptional regulatory function of RNF114 in BC, offering new insights into its oncogenic role and contribution to HER2-positive BC progression.

## Introduction

Breast cancer (BC) is the most prevalent cancer among women worldwide^1^. Despite progress in patient management, BC remains the second leading cause of cancer-related mortality^2^. Based on histopathological criteria including the expression of estrogen receptor (ER), progesterone receptor (PR) and/or human epidermal growth factor receptor 2 (HER2), BC is classified into four major subtypes: Luminal A, Luminal B, HER2-positive, and basal-like^3^. Among them, HER2-positive BC represents 15-20% of BC and is associated with high relapse and poor prognosis^4^, ^5^. Currently, trastuzumab and lapatinib have been approved for HER2-targeted therapy, but many HER2-positive BC metastatic patients either do not respond or experience disease progression within a year despite initial response^6^. Obviously, relying solely on HER2 expression status cannot fully predict the response to HER2-targeted therapy.

RNF114, aka ZNF313, is a recently identified zinc-binding protein with C2H2 and RING-finger structures. RNF114 expression is reduced in azoospermic testes, suggesting a potential role in spermatogenesis and male fertility^7^. *RNF114* was also identified as a novel psoriasis susceptibility gene by genome-wide association study (GWAS), suggesting it is associated with immune related diseases^8^. In addition, *RNF114* resides on human chromosome 20q13.13, a region frequently amplified in various malignancies^9^, ^10^. It has been previously reported that RNF114, a member of the E3 ubiquitin ligase family, plays a critical role in regulating cell cycle progression, differentiation, and senescence^11^-^13^. Previous studies have shown that Nimbolide treatment can inhibit RNF114-based degradation, resulting in trapping of PARP1 and synthetic lethality in BRCA-mutated cancers^14^-^16^. However, it's important to reveal novel functions and interacting molecules of RNF114 in tumor progression. Here, we bridge this knowledge gap by uncovering a novel mechanism through which RNF114 interacts with EWSR1 to enhance *VEGFR2* transcriptional activity, thereby promoting cell proliferation and autophagy via the MEK/ERK signaling pathway.

## Material and Methods

### BC tissue microarray (TMA) and immunohistochemistry staining

Three identical 180-spot, paraffin-embedded, BC TMA (ZL-Brc Sur1801) containing 90 paired BC and adjacent breast tissues were purchased from Weiao Biotech (Shanghai, China) and subjected to immunohistochemistry (IHC) staining for RNF114, EWSR1, and VEGFR2, respectively. The inclusion criteria for patient selection required a confirmed pathological diagnosis of BC, the availability of paired BC and adjacent normal tissue samples, and complete clinical and survival data. Exclusion criteria included IHC staining failure, inaccurate survival data, or the presence of comorbid conditions (e.g., psoriasis, miliaria crystallina, or vascular diseases) that could potentially influence the expression profiles of RNF114, EWSR1 or VEGFR2. Ultimately, 78 paired samples that satisfied these criteria were included in the final analysis. The clinical parameters of the 78 enrolled patients are detailed in Table S1.

The stained tissue microarrays were scanned using Nanozoomer Digital Pathology (Hamamatsu, Japan) and quantified with HALO software (Indica Labs, USA). RNF114 staining scores from HALO included the staining area and mean intensity, adjusted on a centesimal scale. Positive area percentages were categorized as: 0 (0-4%), 1 (5-24%), 2 (25-49%), 3 (50-74%), or 4 (75-100%). Mean intensity scores were classified as follows: 0 (no color reaction), 1 (mild reaction), 2 (moderate reaction), or 3 (intense reaction). Final immunoreactive score was calculated by multiplying the staining area by mean intensity (range 0-12), categorizing samples into low-level RNF114 expression (score 0-4) and high-level RNF114 expression (score 4-12).

### Cell lines

Human BC cell lines SKBR3, BT474, AU565, MCF7, MDA-MB-231 and HCC1937 were purchased from Zhongqiaoxinzhou Biotech (Shanghai, China). Human immortalized normal breast epithelial Hs578Bst cells was purchased from Jennio Biotech (Guangzhou, China). BT474, SKBR3, AU565, and MCF7 cells were cultivated in RPMI1640 medium supplemented with 10% FBS, while other cells were cultivated in DMEM containing 10% FBS in a humidified cell incubator at 37°C and 5% CO_2_.

### Cell model construction

To construct the stable RNF114 knockdown HER2-positive BC cells, RNF114-specific shRNA (5′-CCATGGCTGCCGTAAGAATTT-3′) was synthesized and inserted into vector psi-LVRU6MP (GeneCopoeia Inc.). Lentiviral particles were generated to infect SKBR3 and BT474 cells, and stable RNF114 knockdown cells were selected using puromycin (Solarbio).

### Western blot analysis

Proteins were extracted and quantified, then separated by SDS-PAGE, transferred to PVDF membranes, blocked, and incubated with antibodies for detection. Detailed information can be found in Doc S1, and the primary antibodies used were provided in Table S2.

### RT-PCR and real-time quantitative RT-PCR

RNA extraction and quantification were followed by qRT-PCR analysis using ΔΔCt normalization. Detailed information can be found in Doc S1, and the primers used were listed in Table S3.

### Cell assays for proliferation, migration, and invasion

CCK8, wound healing, and transwell invasion assays were performed to assess cellular proliferation, migration, and invasion capacities, respectively. Detailed information can be found in Doc S1.

### Co-immunoprecipitation

Proteins were immunoprecipitated with a specific antibody and protein A+G agarose beads, then analyzed by Western blot. Detailed information can be found in Doc S1.

### RNA-seq assay

RNA extraction and transcriptome sequencing were conducted on stable RNF114 knockdown SKBR3 cells. Detailed information can be found in Doc S1.

### Transmission electron microscopy (TEM)

TEM was utilized to examine the ultrastructural changes in stable RNF114 knockdown cells. Detailed information can be found in Doc S1.

### Dual fluorescent autophagic flux

Ad-mCherry-GFP-LC3B vectors (Shanghai Genechem Co., Ltd.) were transfected into RNF114 knockdown and control SKBR3 cells. After 48h, live cells were imaged using stochastic optical reconstruction super-resolution microscopy (Nikon, Japan).

### Luciferase activity assay

Different lengths of *VEGFR2* promoter fragments (-2000 to +302bp, -1000 to -501bp, and -951 to -901bp) were synthesized and cloned into the pGL3 basic luciferase reporter vector (WZ Biosciences Inc). These plasmids, along with internal reference pRL-CMV plasmids (Promega), pENTER-RNF114 or pENTER-EWSR1 expression plasmids (WZ Biosciences Inc), were co-transfected into 293T cells. After 48h, luciferase activity was examined following the manufacturer's instructions (Beyotime, China).

### Chromatin immunoprecipitation (ChIP)

ChIP-IT® Express Enzymatic Shearing Kit (Active Motif, USA) was used for the ChIP assay. SKBR3 cells were fixed with 37% formaldehyde solution and collected for chromatin preparation. A portion of the chromatin was used to evaluate shearing efficiency and DNA concentration, while the remaining chromatin was used to configure ChIP reaction system. The precipitated DNA was subsequently amplified using specific qPCR primers (Table S4).

### *In vivo* tumor xenograft model

Four-week-old BALB/c nude mice were purchased from GemPharmatech (Nanjing, China). Stable RNF114 knockdown BT474 cells or shNC BT474 control cells were suspended in 0.2 ml PBS, and subcutaneous injection of 1×10^7^ cells was performed on the right dorsal flank of 5-week-old mice. After 18 days, mice were randomly assigned to four groups (n=3 per group) and administered either Cabozantinib (100 mg/kg) or a CMCNA vehicle orally once daily for 5 days. Tumor diameters were measured every 2 days for 22 days, after which the mice were euthanized. Tumor volume was calculated using the following formula: Volume(mm^3^) = (Length × Width^2^)/2. Tumors were excised and analyzed using IHC. All animal experiments were approved by the Animal Ethics Committee of West China Hospital and conducted according to the institutional and national guidelines.

### Statistical analysis

Data statistics were performed using GraphPad Prism 8. Data were shown as mean ± standard deviation (SD). Comparisons between two groups were analyzed using Student's t-test. Comparisons among multiple groups were conducted using one-way analysis of variance (ANOVA), followed by Tukey's multiple comparisons test when applicable. Spearman analysis was used to assess the correlation between *RNF114* expression and other genes. Survival rates were calculated using the Kaplan-Meier method, with significance assessed by Log-rank test. P-value<0.05 was considered statistically significant.

## Results

### RNF114 is highly expressed and associated with poor prognosis in BC

**Figure 1:**
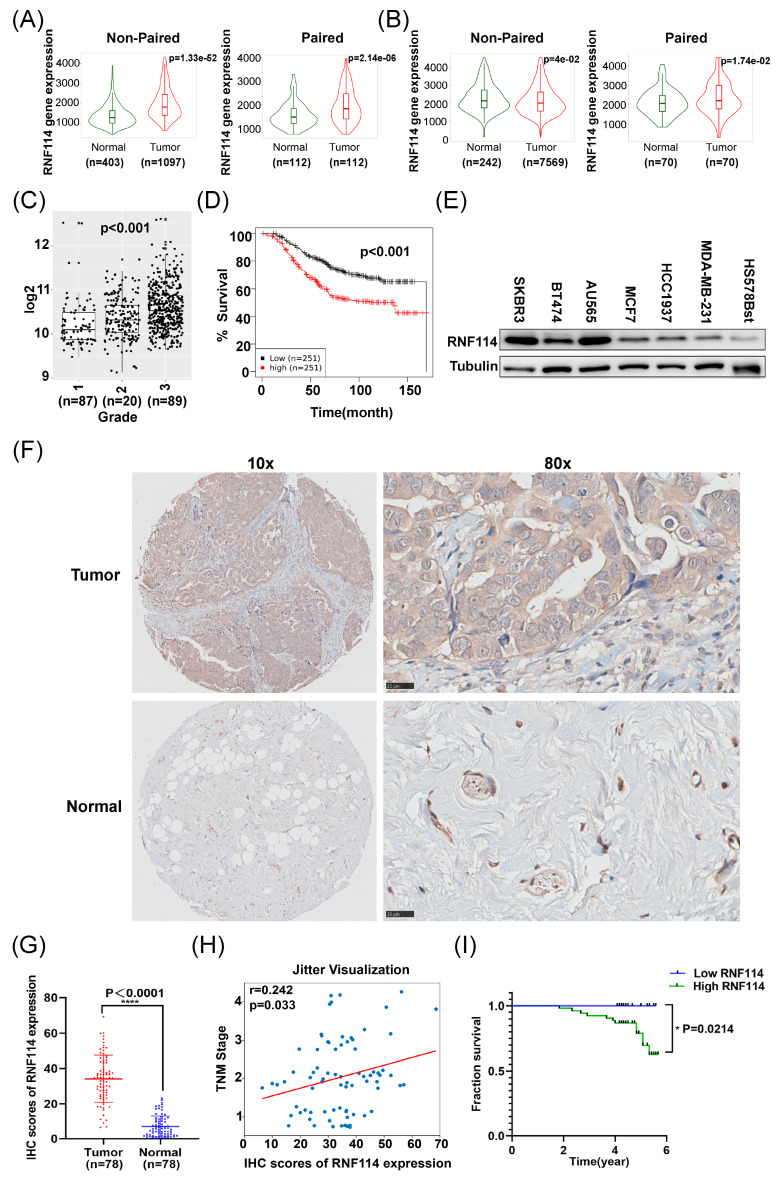
** RNF114 is highly expressed in BC and associated with poor prognosis**. Using RNA-seq (A) and gene chip (B) data from the Kaplan-Meier plotter database, *RNF114* expression in BC and adjacent normal tissues was compared. (C) Analysis of Gent2 database on differential RNF114 expression across BC grades. (D) Analysis of Gent2 database showed elevated RNF114 levels are associated with poor prognosis in BC Patients. (E) BC cell lines (SKBR3, BT474, AU565, MCF-7, HCC1937, MDA-MB-231) and normal breast cell line Hs578Bst were subjected to RNF114 expression analysis using WB. (F) IHC staining of RNF114 in BC and normal breast tissues. Scale bars, 25 μm. (G) IHC analysis showed higher RNF114 expression in BC tissues compared to adjacent normal tissues. (H) Correlation between RNF114 IHC expression scores and TNM stage. The scatter plot shows individual data points with a jittered distribution to enhance visibility. (I) The Kaplan-Meier curve indicated that the high-RNF114 group (n=53) had poorer survival outcomes in BC patients compared to low-RNF114 group (n=25). *P<0.05; ****P<0.0001.

We initially analyzed *RNF114* expression in BC and normal tissues utilizing the Kaplan-Meier plotter database. Both RNA-seq data (Figure 1A) and gene chip data (Figure 1B) showed higher *RNF114* expression in BC compared to adjacent normal tissues, regardless of whether non-paired or paired samples were used as controls. Furthermore, the Gent2 database analysis showed that *RNF114* expression differed across BC grades, with the highest expression in grade 3 (p<0.001) (Figure 1C). Meanwhile, higher *RNF114* levels were also linked to poor prognosis in BC patients (p<0.001) (Figure 1D).

We further examined RNF114 expression in six BC cell lines (SKBR3, BT474, AU565, MCF-7, HCC1937, MDA-MB-231) and a normal breast epithelial cell line (Hs578Bst) using western blot (WB). This result showed significantly higher RNF114 expression in six BC cell lines compared to the normal breast cell line (Figure 1E). Moreover, RNF114 expression was significantly higher in HER2-positive BC cell lines (SKBR3, BT474 and AU565) compared to non-HER2-positive BC cell lines (MCF7, MDA-MB-231 and HCC1937) (Figure 1E).

Additionally, we examined RNF114 expression in BC and adjacent normal tissues using immunohistochemistry, confirming higher RNF114 expression in BC tissues (n=78) (Figure 1F,G). Furthermore, Pearson correlation analysis showed that high expression of RNF114 was significantly positively correlated with TNM stage (r = 0.242, p=0.033) (Figure 1H), but there was no significant correlation with other parameters, such as age and tumor size (p > 0.05) (Figure S1).

To reveal the correlation between RNF114 expression and the prognosis of BC, RNF114 expression was scored by multiplying intensity (0-3) and staining area percentage (0-4). Final scores were analyzed using X-tile software^17^ (Figure S2) and divided into low-RNF114 (score 0-4, n = 25) and high-RNF114 (score 4-12, n =53) groups. Kaplan-Meier survival analysis demonstrated that BC patients with high-RNF114 expression had poorer survival outcomes compared to those with low-RNF114 expression (Figure 1I).

Overall, these results indicate that elevated RNF114 expression in BC is associated with TNM stage and poor prognosis, suggesting that RNF114 may function as a novel biomarker for predicting patient outcomes.

### RNF114 knockdown significantly suppresses proliferation, migration, invasiveness and autophagy in HER2-positive BC cells

**Figure 2:**
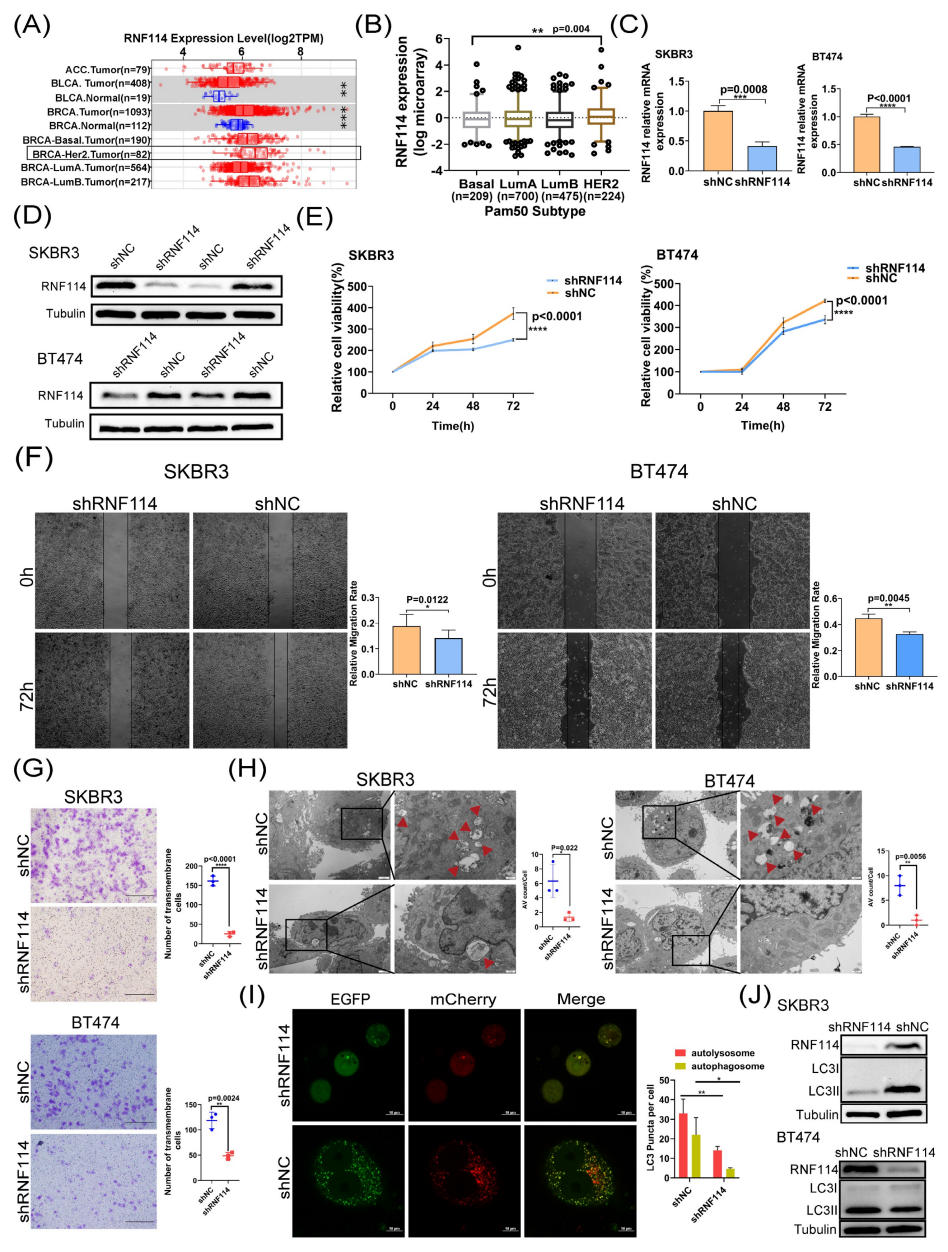
** RNF114 knockdown inhibited proliferation, migration, invasiveness and autophagy in HER2-positive BC cells.** (A) RNF114 expression in BC and adjacent normal tissues from the TIMER database, with the black box highlighting HER2-positive BC. (B) Significant RNF114 expression difference between HER2-positive BC and other subtypes in the METABRIC dataset (cBioPortal). Reduced mRNA (C) and protein levels (D) of RNF114 in stable knockdown SKBR3 and BT474 HER2-positive BC cells. (E) RNF114 knockdown inhibited SKBR3 and BT474 cells proliferation, (F) migration, and (G) invasion. (H) RNF114 knockdown significantly reduced AVs in SKBR3 and BT474 cells. Red arrowheads denoting autophagosomes. Scale bars, 2 μm (original) and 500 nm (magnification). (I) Autophagosomes (yellow dots) and autolysosomes (red dots) were observed in RNF114 knockdown SKBR3 cells. Scale bars, 10 µm. (J) LC3II levels were reduced in RNF114 knockdown SKBR3 and BT474 cells. All data are presented as mean ± SD. *P<0.05; **P < 0.01; ***P<0.001; ****P<0.0001.

TIMER database analysis showed higher RNF114 expression in HER2-positive BC compared to other BC subtypes (Figure 2A). Additionally, the consistent result was also observed in the METABRIC dataset (http://www.cbioportal.org/), showing significantly higher RNF114 expression in HER2-positive BC compared to other subtypes (p=0.004) (Figure 2B). Given higher RNF114 expression in HER2-positive BC, we focused towards exploring its potential role in this subtype in our subsequent research. We established two stable RNF114 knockdown models in SKBR3 and BT474 HER2-positive BC cell lines. qPCR and WB confirmed decreased *RNF114* mRNA and protein levels in these cell models (Figure 2C,D). We further investigated the cellular characteristics of stable RNF114 knockdown HER2-positive BC cells, including proliferation, migration and invasiveness. These results demonstrated that RNF114 knockdown significantly inhibited proliferation (Figure 2E), migration (Figure 2F) and invasion (Figure 2G) in SKBR3 and BT474 cells.

Additionally, analysis of BC transcriptomic data from the TCGA database revealed a significant positive correlation between RNF114 expression and several autophagy-related genes, including *ATG3*,* ATG5*, *ATG7*, *ATG12*, *LC3B*, and *ULK1* (p < 0.05) (Figure S3A-F). Furthermore, TEM analysis revealed a reduced number of autophagic vacuoles (Avs) in RNF114 knockdown SKBR3 and BT474 cells (Figure 2H), indicating that RNF114 knockdown inhibited autophagy in HER2-positive BC cells. Moreover, confocal microscopy of RNF114 knockdown SKBR3 cells transfected with mCherry-GFP-LC3 vectors demonstrated a significant reduction in both yellow and red dots, indicating decreased autophagosome and autolysosome formation (Figure 2I). WB further confirmed a significant reduction in LC3-II, a marker of closed autophagosomes, in RNF114 knockdown SKBR3 and BT474 cells (Figure 2J), indicating inhibited autophagy due to RNF114 downregulation. Collectively, these findings indicated that RNF114 knockdown inhibited the autophagy in HER2-positive BC cells.

### Prediction of RNF114 regulatory pathway in HER2-positive BC cells

**Figure 3:**
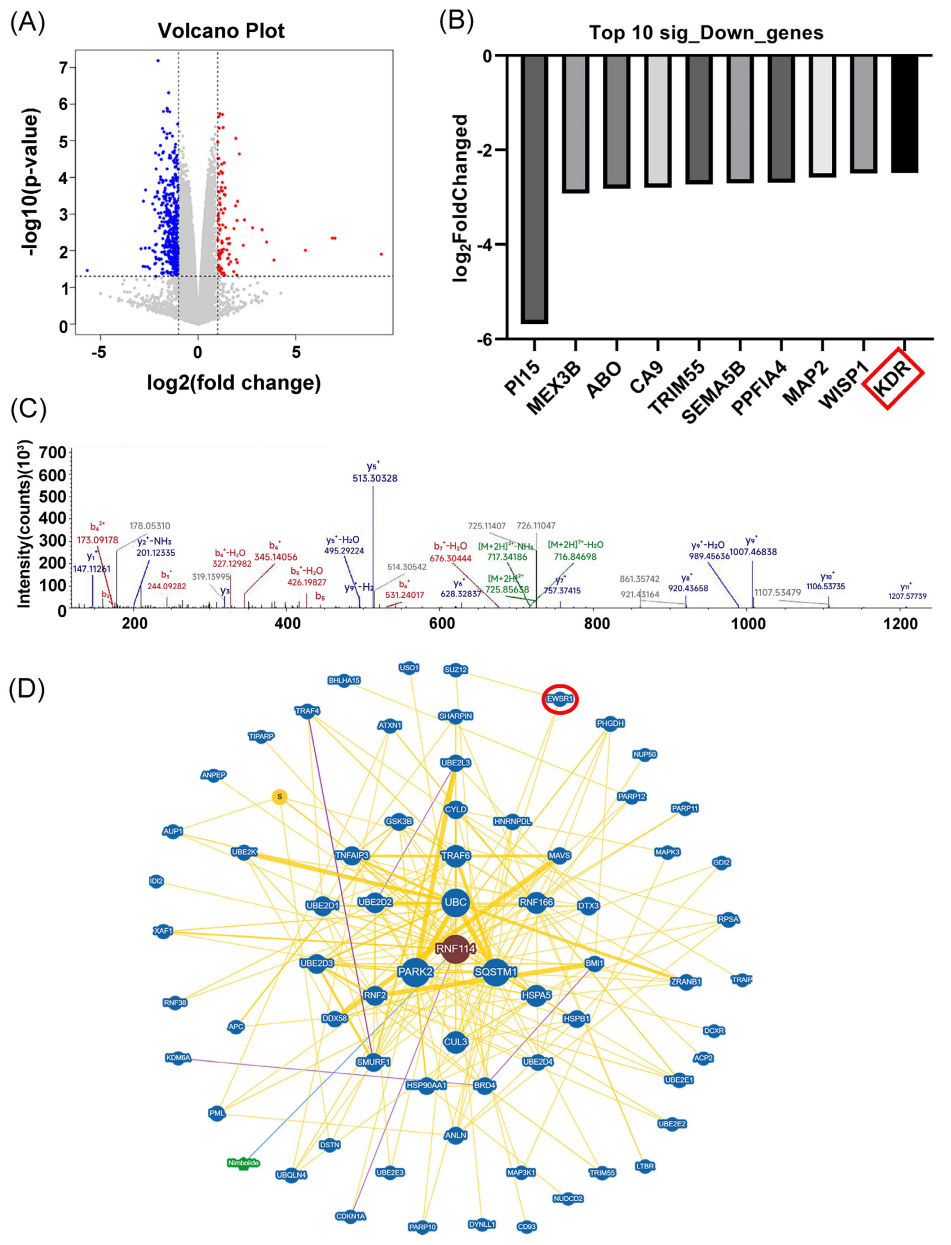
** Transcriptome sequencing and IP-MS predicted RNF114 regulatory pathway in HER2-positive BC cells.** (A) Volcano plot of DEGs. (B) Bar chart showing the top 10 significantly downregulated genes, with* KDR* (*VEGFR2*) highlighted in red box. (C) IP-MS analysis identified EWSR1, with peak peptide GDATVSYEDPPTAK. (D) BioGRID database predicted EWSR1 as a potential RNF114 interaction protein.

To further predict the potential RNF114 regulatory pathway in HER2-positive BC cells, transcriptome sequencing was conducted to identify differentially expressed genes (DEGs) following RNF114 knockdown in SKBR3 cells (Figure 3A). GO enrichment analysis of 482 significantly downregulated genes suggested their involvement in phospholipid metabolic process and regulation of autophagy (Figure S4A). KEGG pathway enrichment showed their association with key pathways such as Ras, PPAR, PI3K-Akt, and focal adhesion (Figure S4B). We further screened the top 10 significantly downregulated genes and found that RNF114 knockdown significantly downregulated *VEGFR2* (*KDR*) expression (log FC=-2.478) (Figure 3B), suggesting *VEGFR2* as a potential RNF114 regulation target.

Furthermore, IP-MS analysis identified EWSR1 as a potential RNF114 interacting protein, with the specific peptide segment GDATVSYEDPPTAK showing the peak value (Figure 3C). Protein-protein interaction (PPI) prediction from BioGRID database also indicated EWSR1 as a potential RNF114 interacting protein (Figure 3D). Additionally, Animal TFDB database (http://bioinfo.life.hust.edu.cn/AnimalTFDB/#!/) predictions indicated EWSR1 can regulate *VEGFR2* transcription with 24 potential binding sites (Table S5). Taken together, these results led us to hypothesize an RNF114/EWSR1/VEGFR2 regulatory axis in HER2-positive BC cells.

### RNF114 is a positive regulator of the VEGFR2 in HER2-positive BC cells

**Figure 4:**
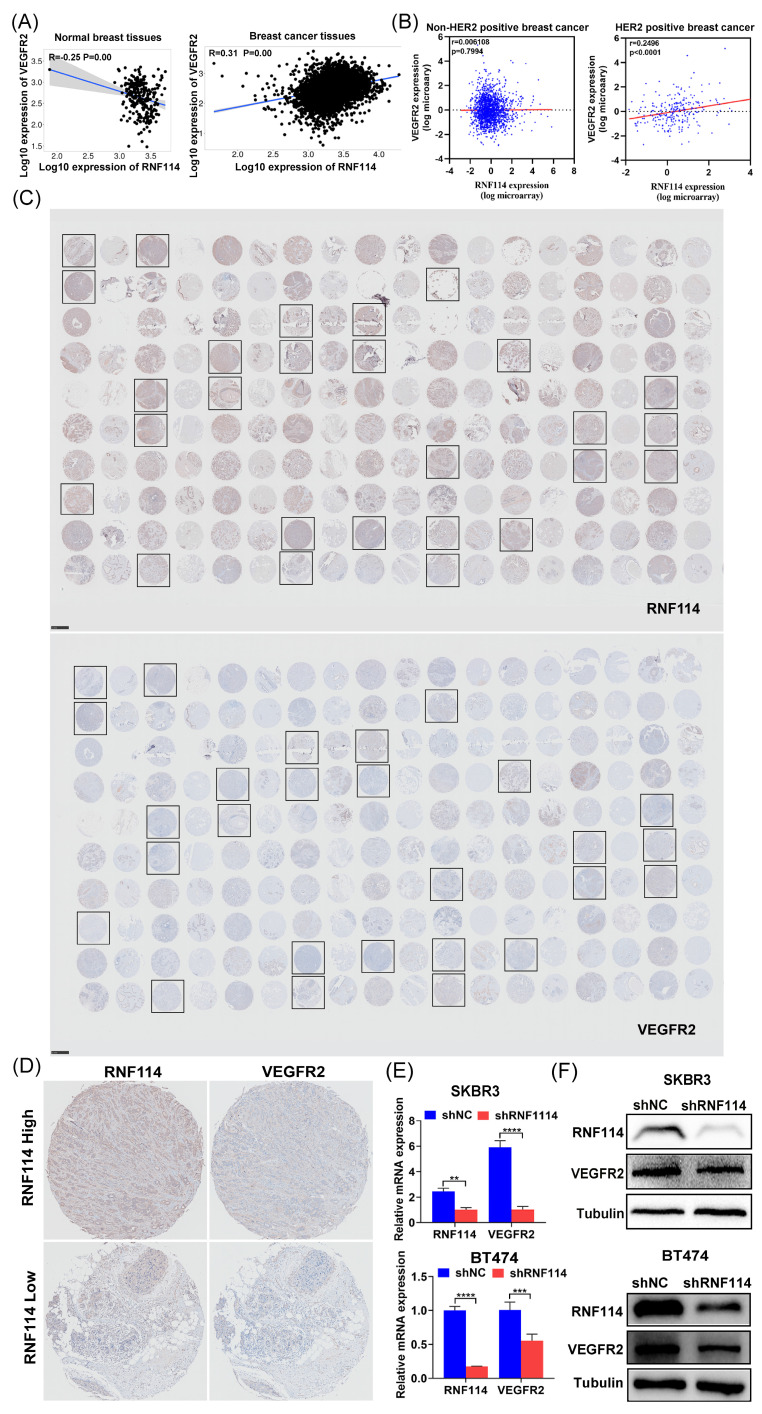
**RNF114 positively regulated VEGFR2 expression in HER2-positive BC cells.** (A) Spearman correlation analysis of RNF114 and VEGFR2 in normal breast and BC tissues using Kaplan-Meier plotter database. (B) Pearson correlation analysis of RNF114 and VEGFR2 in HER2-positive and non-HER2-positive BC tissues using METABRIC dataset. (C) IHC staining of RNF114 and VEGFR2 in 90 paired BC and adjacent tissues, with black frames highlighting HER2-positive BC patients. (D) RNF114 and VEGFR2 immunoreactivity in the same HER2-positive BC patients. *VEGFR2* mRNA (E) and protein expression (F) were reduced in stable RNF114 knockdown HER2-positive BC cell lines.

Kaplan-Meier plotter database analysis showed that* RNF114* and *VEGFR2* were negatively correlated in normal breast tissues (n=242) but positively correlated in BC tissues (n=7569) (Figure 4A). Interestingly, further analysis using METABRIC database revealed a significant positive correlation between *RNF114* and *VEGFR2* in HER2-positive BC patients (n=247), but not in non-HER2-positive subtypes (n=1733) (Figure 4B), indicating that the role of *RNF114* involving* VEGFR2* in BC may be specific to the HER2-positive subtype. BC tissue microarrays (TMAs) were further utilized to confirm the relationship between RNF114 and VEGFR2 in HER2-positive BC. Figure 4C shows IHC staining results for RNF114 and VEGFR2 expression in HER2-positive BC patients (n=27). Figure 4D shows high and low RNF114 and VEGFR2 expression in the same HER2-positive BC patients, indicating a positive correlation between them. Additionally, qPCR and WB analysis showed that RNF114 knockdown reduced both *VEGFR2* mRNA levels (Figure 4E) and protein expression (Figure 4F) in SKBR3 and BT474 cells. These results suggested that RNF114 functioned as a positive regulator of VEGFR2 in HER2-positive BC cells.

### RNF114 interacts with EWSR1 protein in HER2-positive BC cells

**Figure 5:**
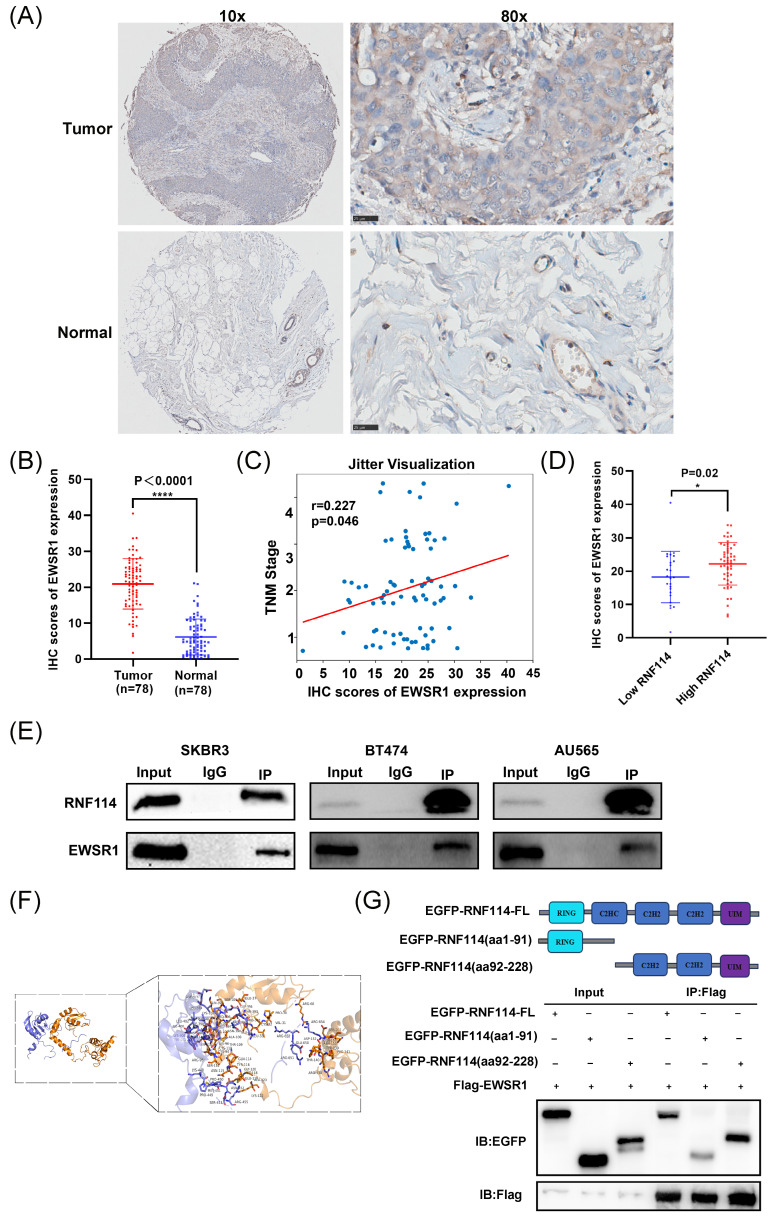
**The endogenous interaction between RNF114 and EWSR1.** (A, B) Representative images of EWSR1 IHC staining in BC and paracancerous tissue. Scale bars, 25 μm. Histogram displays the differences in IHC scores between the two groups. (C) Correlation between EWSR1 IHC expression scores and TNM stage. The scatter plot displays individual data points with jittered distribution for improved visualization. (D) EWSR1 was significantly elevated in the RNF114-high group (n=53) compared to the RNF114-low group (n=25). (E) Co-IP confirmed the endogenous interaction between RNF114 and EWSR1 in three HER2-positive BC cells (SKBR3, BT474 and AU565). (F) Homologous modeling and molecular docking of RNF114 (brown ribbon) and EWSR1 (purple-blue ribbon). Rod-shaped structures represent interacting amino acid residues. (G) Co-IP of Flag-EWSR1 and EGFP-RNF114 domains. *P < 0.05; ****P < 0.0001.

Given that IP-MS identified EWSR1 as a potential RNF114 interacting protein, we firstly conducted IHC analysis of EWSR1 using BC TMA. The results demonstrated significantly higher EWSR1 expression in BC tissues compared to adjacent tissues (Figure 5A,B). Furthermore, Pearson correlation analysis showed that high expression of EWSR1 was significantly positively correlated with TNM stage (r = 0.227, p=0.046) (Figure 5C), but there was no significant correlation with other parameters, such as age and tumor size (p > 0.05) (Figure S5). Additionally, EWSR1 expression was significantly elevated in the RNF114-high group compared to the RNF114-low group (Figure 5D), suggesting a positive correlation between RNF114 and EWSR1 in BC patients. Furthermore, Co-IP assay confirmed endogenous interaction between RNF114 and EWSR1 in three HER2-positive BC cell lines (Figure 5E). Additionally, homologous modeling and molecular docking prediction showed that EWSR1 can bind to RNF114 at CYS-94, GLN-114, ASP-152, SER-202, and other sites, primarily within the aa92-228 domain (Figure 5F). Co-IP of Flag-EWSR1 and EGFP-RNF114 domains further validated this prediction, showing that EWSR1 endogenously bound to RNF114 via its aa92-228 domain (Figure 5G).

### RNF114 interacts with EWSR1 to regulate *VEGFR2* transcription

**Figure 6:**
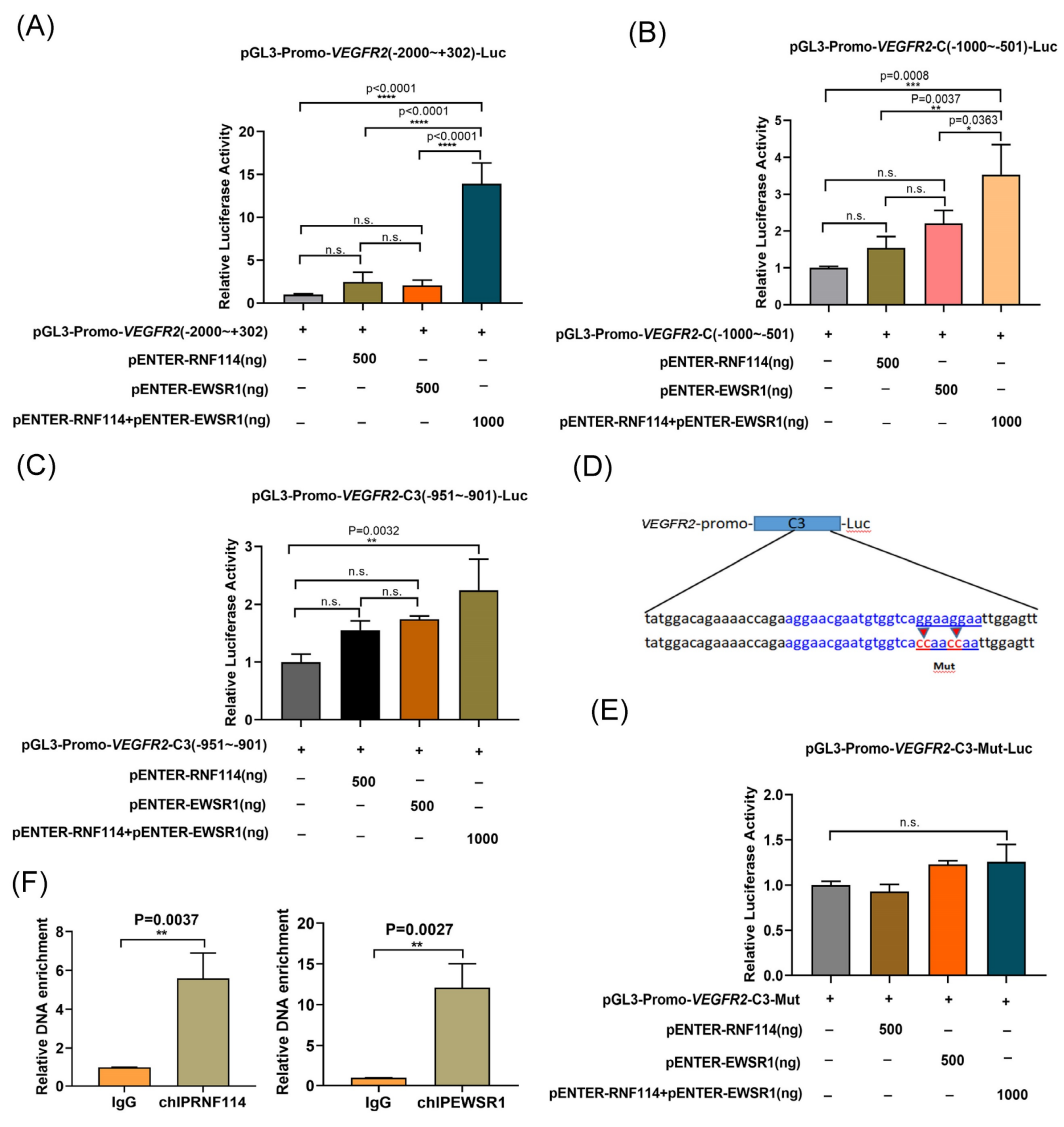
**RNF114 and EWSR1 interaction enhanced *VEGFR2* transcriptional activity.** Co-transfection of RNF114 and EWSR1 significantly increased luciferase activity in *VEGFR2* promoter (-2000bp to +302bp) construct (A), *VEGFR2* promoter-C (-1000bp to -501bp) construct (B), and *VEGFR2* promoter-C3 (-951bp~-901 bp) constructs (C). (D) The mut*VEGFR2*-C3 promoter sequence (-916bp~-909bp 5'-GGAAGGAA -3' mutated to 5'-CCAACCAA-3') was constructed, with the blue-marked region indicating EWSR1 target sequence predicted by the Animal TFDB database. (E) Co-transfection of RNF114 and EWSR1 did not significantly enhance luciferase activity in the mut*VEGFR2*-C3 promoter construct. (F) ChIP-qPCR assays confirmed the predicted site in *VEGFR2* promoter in SKBR3 cells. *P<0.05; **P < 0.01; ***P<0.001; ****P<0.0001; n.s. means no significant difference.

EWSR1 has been reported to act as a potent transcriptional cofactor involved in transcription regulation^18^. Based on this potential RNF114/EWSR1/VEGFR2 regulatory axis, we further investigate* VEGFR2* transcriptional regulatory mechanism. The *VEGFR2* promoter region (-2000bp~+302bp) was subcloned into the pGL3 luciferase reporter plasmid for dual-luciferase analysis. Luciferase reporter assays showed that co-transfection of RNF114 and EWSR1 significantly increased luciferase expression (Figure 6A). Shortening *VEGFR2* promoter to the *VEGFR2*-C fragment (-1000bp~-501bp) produced similar results (Figure 6B). Further truncation to the *VEGFR2*-C3 fragment (-951bp~-901bp) also showed increased luciferase activity with RNF114 and EWSR1 co-transfection (Figure 6C). Furthermore, mut*VEGFR2*-C3 was generated by mutating the GGAAGGAA (-916bp~-909bp) into the CCAAACCAA (Figure 6D). The luciferase reporter assay demonstrated that co-transfection with RNF114 and EWSR1 failed to enhance the luciferase activity of the mut*VEGFR2*-C3 promoter construct (Figure 6E), implying that this mutation disrupted the regulatory function of RNF114 and EWSR1-interacting proteins on *VEGFR2* promoter activity. Additionally, ChIP-qPCR assay demonstrated that RNF114 and EWSR1 can bind to the *VEGFR2* promoter region (-951bp~-901bp) (Figure 6F). Taken together, these findings suggested that RNF114 interacting with EWSR1 can bind to the -951bp~-901bp site of *VEGFR2* promoter and enhance its transcriptional activity.

### RNF114-stimulated VEGFR2 can regulate MEK/ERK pathway and upregulate cell proliferation

**Figure 7:**
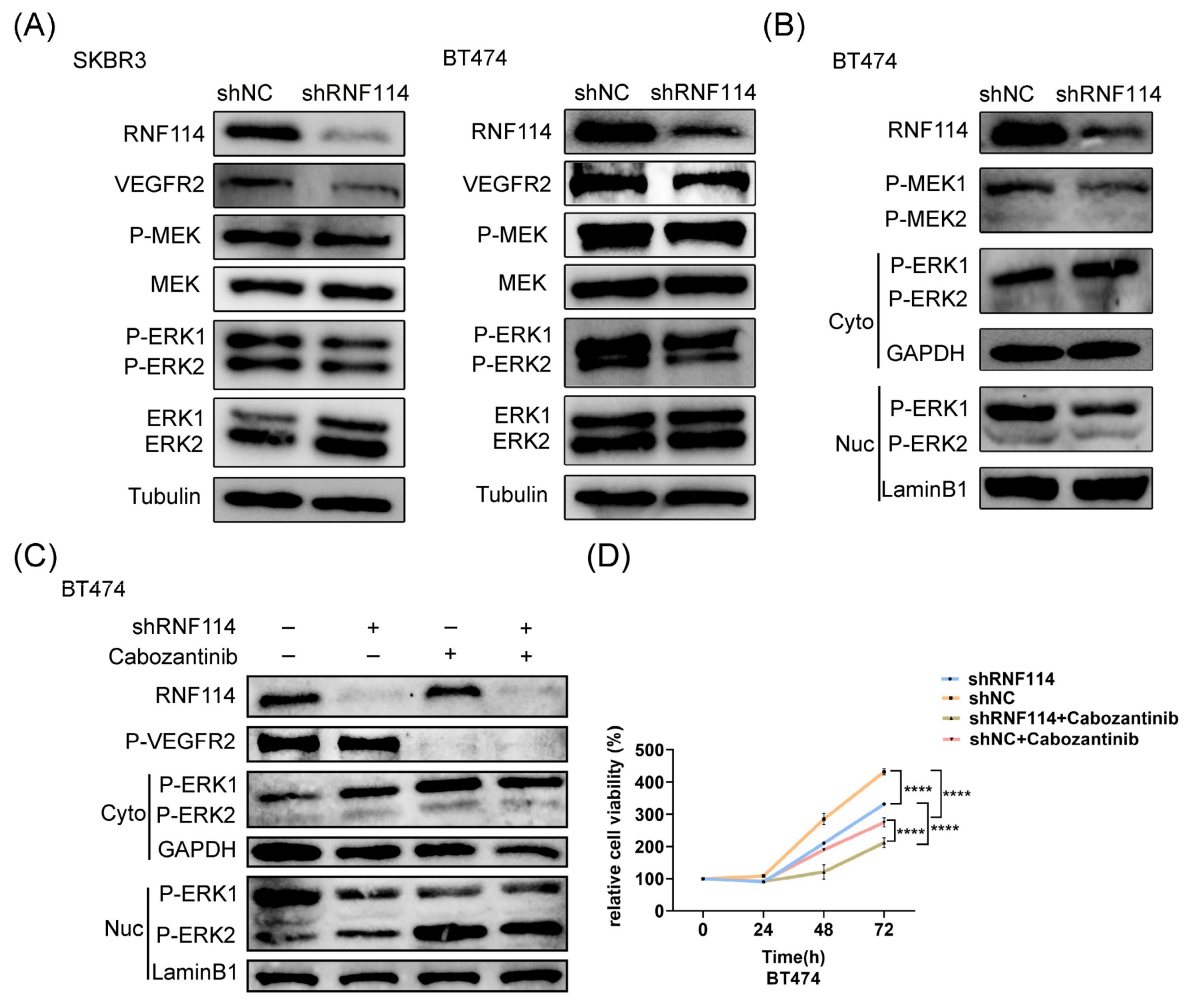
**RNF114-stimulated VEGFR2 regulates MEK/ERK pathway and upregulates cell proliferation in HER2-positive BC cells.** (A) RNF114 knockdown reduced VEGFR2, P-MEK, and P-ERK1/2 expression in SKBR3 and BT474 cells. (B) The nuclear-cytoplasmic separation assay was used to detect P-ERK1/2 translocation, with GAPDH and LaminB1 as internal controls. (C) Stable RNF114 knockdown BT474 cells were treated with 20μM VEGFR2 inhibitor Cabozantinib for 24h before nuclear/cytoplasmic fractionation. (D) CCK8 assay demonstrated varying proliferation rates of BT474 cells under different treatment conditions. ****P<0.0001.

WB analysis showed a significant decrease in VEGFR2 protein levels, accompanied by a marked expression reduction in downstream signaling regulators, including P-MEK and P-ERK, in stable RNF114 knockdown cell lines (Figure 7A). Furthermore, the nuclear translocation of P-ERK1/2 was analyzed using a nuclear-cytoplasmic separation assay. This result demonstrated that RNF114 knockdown significantly increased P-ERK1 levels in the cytoplasm while decreasing P-ERK1 levels in the nucleus, suggesting that RNF114 knockdown inhibited P-ERK1 nuclear translocation (Figure 7B). This observation was abolished in BT474 cells treated with 20μM VEGFR2 inhibitor Cabozantinib for 24h (Figure 7C), implying that RNF114 knockdown suppressed P-ERK1 nuclear translocation in a VEGFR2-dependent manner. Additionally, CCK8 analysis showed that combining RNF114 knockdown with VEGFR2 inhibitor Cabozantinib significantly inhibited BT474 cell proliferation (Figure 7D). These results indicated that RNF114-stimulated VEGFR2 regulated MEK/ERK pathway and upregulated cell proliferation in HER2-positive BC cells.

### RNF114 promotes cell autophagy via VEGFR2-RAS-ERK-mTOR pathway

**Figure 8:**
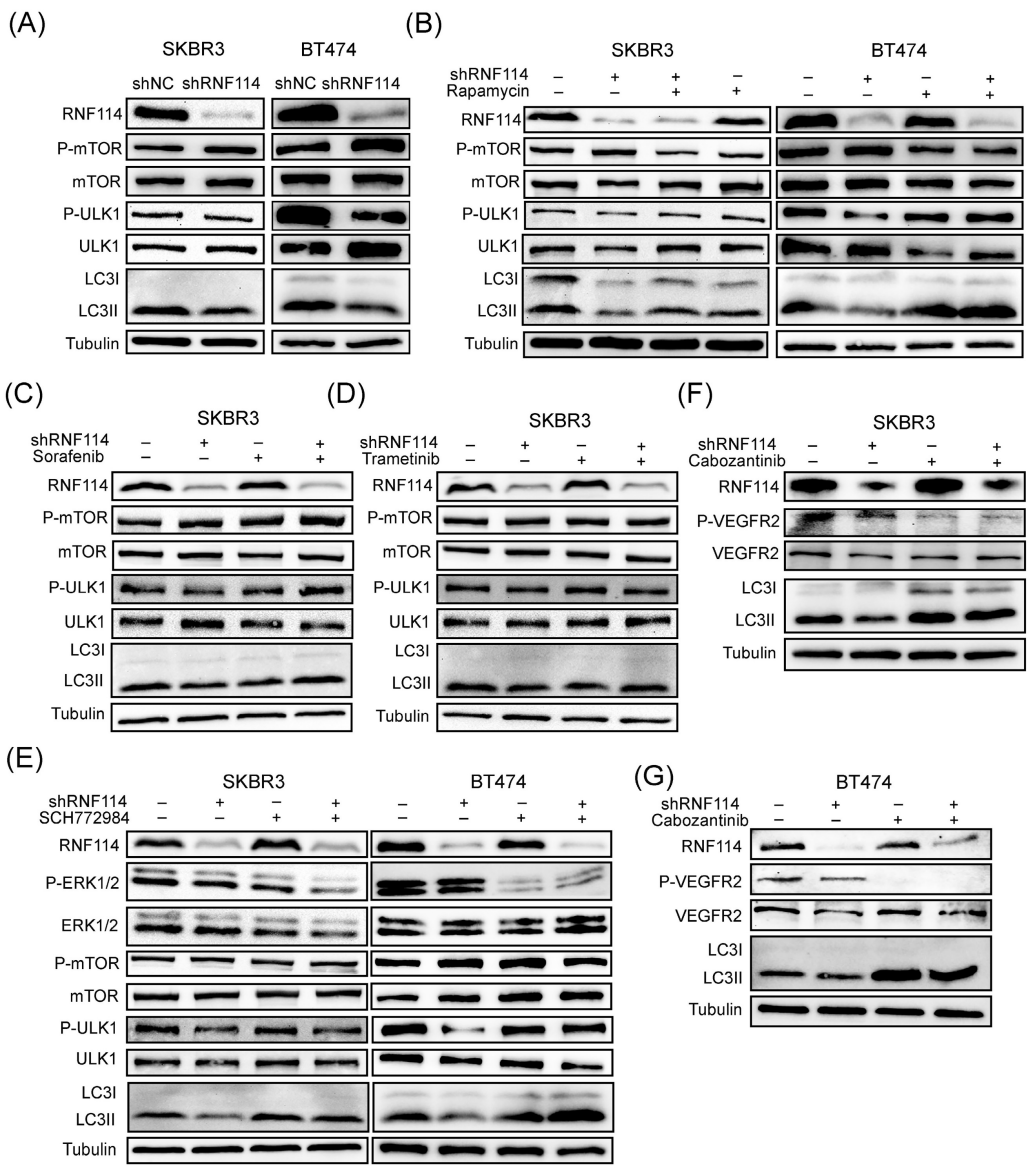
** RNF114-stimulated VEGFR2 can regulate MEK/ERK pathway and upregulate cell autophagy.** (A) P-mTOR and P-ULK1 in the mTOR pathway were detected in stable RNF114 knockdown SKBR3 and BT474 cells. (B) RNF114 knockdown-induced autophagy suppression can be rescued by 24h treatment with 100 nM rapamycin in SKBR3 and BT474 cells. The reduction in LC3-II expression levels caused by RNF114 knockdown can be rescued by the RAF inhibitor Sorafenib (C), the MEK inhibitor Trametinib (D), the ERK inhibitor SCH772984 (E), and VEGFR2 inhibitor Cabozantinib (F, G), respectively.

To clarify the mechanism by which RNF114 regulated autophagy, WB analysis was performed to assess its effect on the classical mTOR pathway. We found that RNF114 knockdown increased mTOR phosphorylation while suppressing ULK1 phosphorylation (Figure 8A). Autophagy suppression resulting from RNF114 knockdown can be rescued by 24-hour treatment with 100 nM rapamycin, suggesting that RNF114 induced autophagy by suppressing mTOR pathway (Figure 8B). Furthermore, RNF114 knockdown reduced LC3-II levels, which was rescued by the RAF inhibitor Sorafenib, the MEK inhibitor Trametinib and the ERK inhibitor SCH772984, respectively (Figure 8C-E). Additionally, the autophagy suppression induced by RNF114 knockdown can be rescued by treatment with VEGFR2 inhibitor Cabozantinib in HER2-positive SKBR3 and BT474 cells (Figure 8F,G). These findings indicated that RNF114-stimulated VEGFR2 can regulate MEK/ERK pathway and upregulate cell autophagy.

### RNF114 knockdown significantly suppresses tumor growth and autophagy *in vivo*

**Figure 9:**
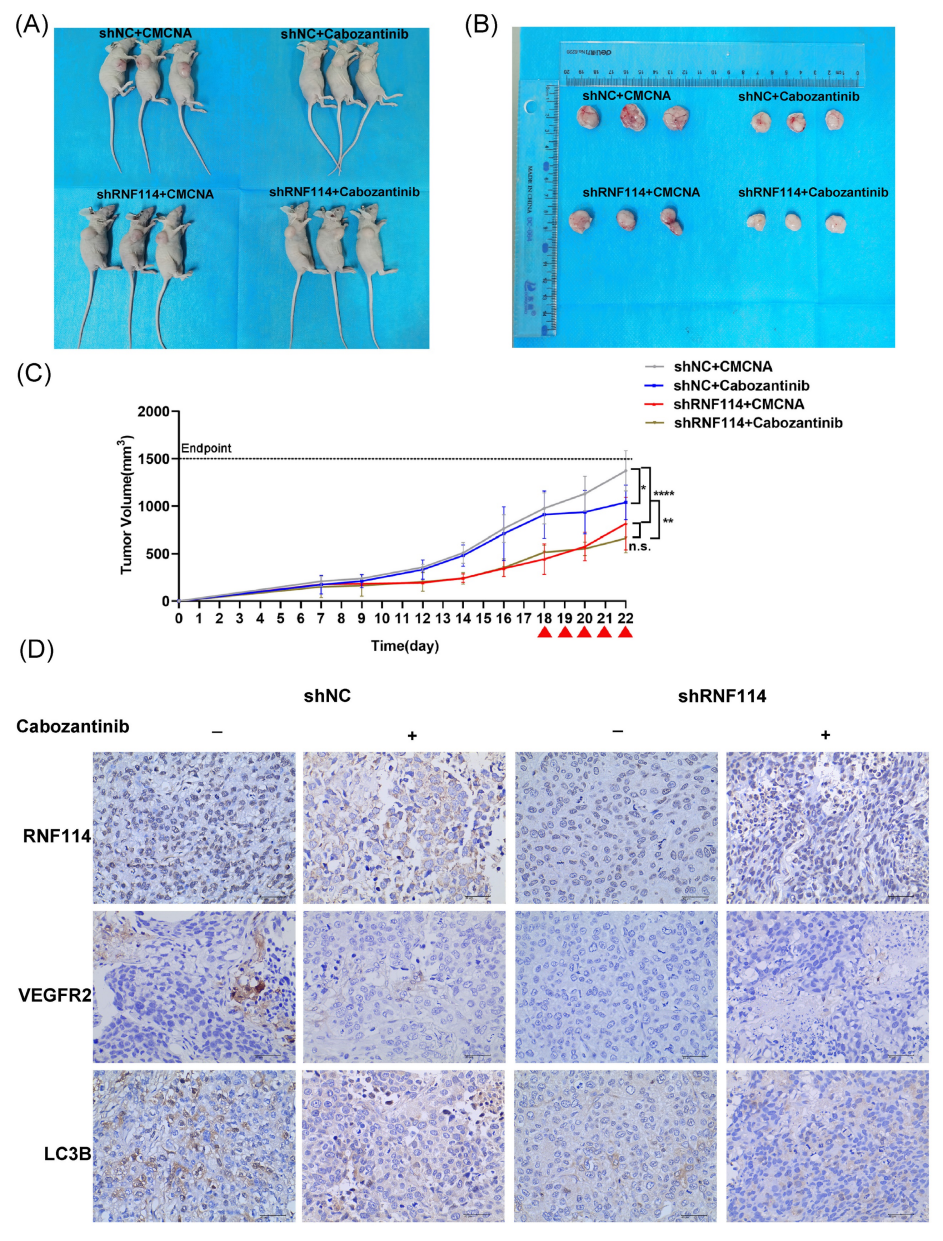
**RNF114 promotes tumor growth in the BT474 xenograft model.** (A) Photos of three mice from each group. (B) Photos of three tumor tissues collected from each group for analysis. (C) Tumor growth rate was assessed by calculating the volume using the formula: Volume (mm³) = (Length × Width²) / 2. Red arrow, orally administered Cabozantinib (100 mg/kg). n.s., no significance; *P < 0.05; **P < 0.01; ***P < 0.001; ****P < 0.0001. (D) IHC staining was conducted for RNF114, VEGFR2, and LC3B on xenograft tumor tissues. Scale bar, 40 µm.

To investigate the role of RNF114 in HER2-positive BC *in vivo*, stable RNF114 knockdown BT474 cells were subcutaneously injected into 5-week-old nude mice, with shNC cells as a control. Eighteen days after implantation, tumor-bearing mice were randomly assigned to four groups and treated orally once daily with either Cabozantinib or CMCNA as a control. Our results showed a 41% reduction in average tumor volume in mice injected with RNF114 knockdown cells compared to those injected with shNC cells (p <0.0001). Notably, treatment with Cabozantinib significantly decreased average tumor volume in mice implanted with shNC BT474 cells (p =0.0143), but no significant difference in mice implanted with RNF114 knockdown cells (p = 0.4869) (Figure 9A-C). These results suggested that RNF114 promoted the growth of HER2-positive BC cells in a VEGFR2-dependent manner *in vivo*. Furthermore, IHC analysis of LC3B staining indicated a downregulation in tumors derived from stable RNF114 knockdown BT474 cells compared to shNC BT474 cells. Oral Cabozantinib treatment partially abolished this difference (Figure 9D).

## Discussion

HER2, aka ERBB2, is a member of the ErbB tyrosine kinase receptor family^19^. It is involved in processes such as cell growth, activation, and proliferation by activating downstream signaling pathways, including the PI3K/AKT and RAS/RAF/MEK/ERK pathways^19^, ^20^. HER2-positive BC is characterized by high malignancy, a significant risk of recurrence, and a strong propensity for metastasis^5^, ^21^. The development of precise diagnostic methods, as well as targeted drug development and application for HER2-positive BC, is of great significance. Previous studies have revealed that HER2 signaling is associated with angiogenesis regulated by vascular endothelial growth factor (VEGF) and its receptor (VEGFR)^22^, ^23^. In HER2-positive BC, VEGFR2 expression are significantly higher compared to other subtypes^24^, ^25^. Combining HER2-targeted therapies with VEGFR2 inhibitors has been shown to suppress tumor resistance^26^, ^27^ and significantly improve overall survival rates in HER2-positive BC patients^28^. These findings indicate that VEGFR2 is a crucial therapeutic target in HER2-positive BC. However, the regulatory mechanisms of VEGFR2 in HER2-positive BC remain to be fully elucidated.

Our current research showed that RNF114 is highly expressed in both BC tissues and established BC cell lines, and its elevated expression correlates with TNM stage and poor survival outcomes (Figure 1), which could be utilized as a novel biomarker for predicting BC patient outcomes. Interestingly, the expression of RNF114 is significantly higher in HER2-positive BC, and knockdown of RNF114 significantly inhibits cell proliferation, migration, invasiveness, and autophagy in HER2-positive BC cells, revealing the potent roles of RNF114 in HER2-positive BC (Figure 2). Transcriptome sequencing suggested that *VEGFR2* is a candidate target of RNF114 in HER2-positive BC (Figure 3A,B) and our further experiments confirmed that RNF114 is a positive regulator of VEGFR2 in HER2-positive BC cells (Figure 4).

Notably, IP-MS results showed EWSR1, which may regulate *VEGFR2* transcription, is a candidate RNF114 interacting protein (Figure 3C,D). EWSR1 is a multifunctional protein involving gene expression, RNA processing, cell differentiation, autophagy, and mitosis regulation^29^, ^30^. Although gene fusions of EWSR1 occur in multiple sarcomas^31^-^35^, the role of wild-type EWSR1 in tumors remains poorly understood. Our study demonstrated a significantly higher expression level of EWSR1 in BC, and its elevated expression correlates with TNM stage, indicating its potential as a clinical biomarker. Additionally, we showed that EWSR1 is positively correlated with RNF114, and endogenously binds to RNF114 via its aa92-228 domain in HER2-positive BC (Figure 5). This complex can bind to -951~-901bp site of *VEGFR2* promoter, and enhance its transcriptional activity (Figure 6). These findings revealed the regulatory mechanisms of VEGFR2 in HER2-positive BC, while also expanding the known roles of EWSR1 beyond its involvement in sarcoma-related gene fusions.

Our study further suggested that RNF114-stimulated VEGFR2 can regulate MEK/ERK pathway and upregulate cell proliferation (Figure 7) and autophagy (Figure 8) in HER2-positive BC. In accordance with these findings, the mouse xenograft model demonstrated that RNF114 can promote HER2-positive BC growth in a VEGFR2-dependent manner (Figure 9).

**Figure 10:**
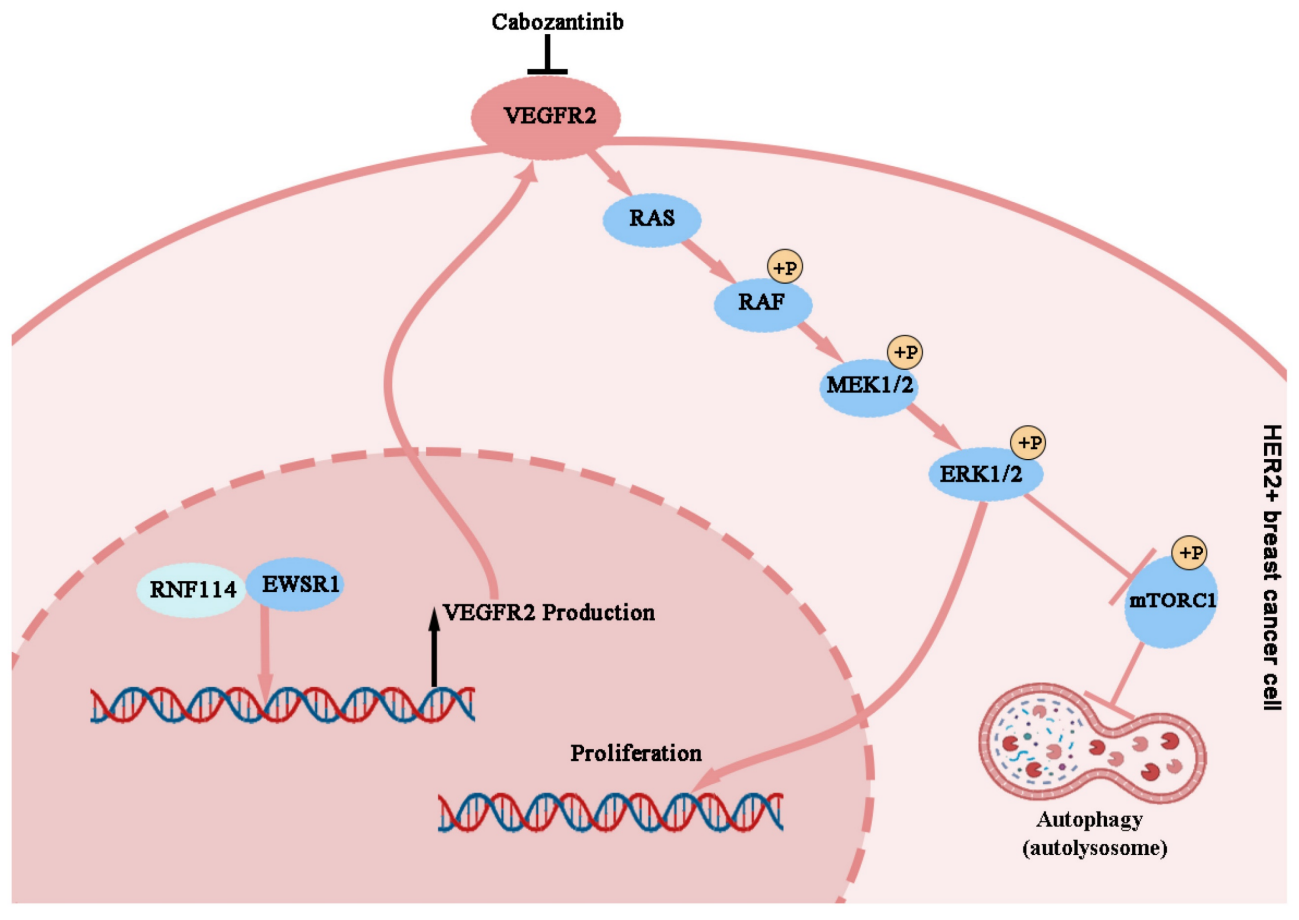
Working model of RNF114 regulating VEGFR2 expression and further affecting proliferation and autophagy in HER2-positive BC cells.

In summary, our study demonstrated that RNF114 interacts with EWSR1 to bind to the -951~ -901 bp site of the *VEGFR2* promoter, enhancing its transcriptional activity. And RNF114-stimulated VEGFR2 can promote cell proliferation and autophagy in HER2-positive BC cells via regulating MEK/ERK pathway (Figure 10).

In previous studies, RNF114 was suggested as a potential target in BRCA-mutated breast cancer for its E3 ligase activity inducing protein degradation^14^, ^15^. Here we first described the transcriptional regulatory pathway (RNF114-EWSR1-VEGFR2) through which RNF114 promotes tumor proliferation and autophagy in HER2-positive BC. Our study expands the understanding of RNF114 in transcriptional regulation by demonstrating that RNF114 regulates *VEGFR2* transcription through interplay with its cofactor EWSR1. These findings enrich the understanding of RNF114's functions, while also expanding the known roles of EWSR1 beyond its involvement in sarcoma-related gene fusions. Furthermore, our findings provide novel insights into the oncogenic role of RNF114 and its contribution to HER2-positive BC progression.

## Supplementary Material

Supplementary methods, figures and tables.

## Abbreviations

BC: Breast cancer
ER: Estrogen receptor
PR: Progesterone receptor
GWAS: Genome-wide association study
GO: Gene Ontology
KEGG: Kyoto Encyclopedia of Genes and Genomes
HER2: Human epidermal growth factor receptor 2
VEGFR2: Vascular endothelial growth factor receptor 2
EWSR1: EWS RNA-binding protein 1

## References

[1] Siegel RL, Miller KD, Fuchs HE, Jemal A (2022). Cancer statistics, 2022. CA: a cancer journal for clinicians.

[2] Hong R, Xu B (2022). Breast cancer: an up-to-date review and future perspectives. Cancer Commun (Lond).

[3] Banerji S, Cibulskis K, Rangel-Escareno C, Brown KK, Carter SL, Frederick AM (2012). Sequence analysis of mutations and translocations across breast cancer subtypes. Nature.

[4] Taurelli Salimbeni B, Ferraro E, Boscolo Bielo L, Curigliano G (2023). Innovative Therapeutic Approaches for Patients with HER2-Positive Breast Cancer. Cancer treatment and research.

[5] Slamon DJ, Clark GM, Wong SG, Levin WJ, Ullrich A, McGuire WL (1987). Human breast cancer: correlation of relapse and survival with amplification of the HER-2/neu oncogene. Science (New York, NY).

[6] Nahta R, Shabaya S, Ozbay T, Rowe DL (2009). Personalizing HER2-targeted therapy in metastatic breast cancer beyond HER2 status: what we have learned from clinical specimens. Current pharmacogenomics and personalized medicine.

[7] Ma YX, Zhang SZ, Hou YP, Huang XL, Wu QQ, Sun Y (2003). Identification of a novel human zinc finger protein gene ZNF313. Acta biochimica et biophysica Sinica.

[8] Capon F, Bijlmakers MJ, Wolf N, Quaranta M, Huffmeier U, Allen M (2008). Identification of ZNF313/RNF114 as a novel psoriasis susceptibility gene. Hum Mol Genet.

[9] Wang D, Zhu ZZ, Jiang H, Zhu J, Cong WM, Wen BJ (2015). Multiple genes identified as targets for 20q13.12-13.33 gain contributing to unfavorable clinical outcomes in patients with hepatocellular carcinoma. Hepatol Int.

[10] Narayan G, Murty VV (2010). Integrative genomic approaches in cervical cancer: implications for molecular pathogenesis. Future Oncol.

[11] Han J, Kim YL, Lee KW, Her NG, Ha TK, Yoon S (2013). ZNF313 is a novel cell cycle activator with an E3 ligase activity inhibiting cellular senescence by destabilizing p21(WAF1.). Cell Death Differ.

[12] Huang S, Li Y, Yuan X, Zhao M, Wang J, Li Y (2019). The UbL-UBA Ubiquilin4 protein functions as a tumor suppressor in gastric cancer by p53-dependent and p53-independent regulation of p21. Cell Death Differ.

[13] Xia J, Ma N, Shi Q, Liu QC, Zhang W, Cao HJ (2024). XAF1 promotes colorectal cancer metastasis via VCP-RNF114-JUP axis. J Cell Biol.

[14] Spradlin JN, Hu X, Ward CC, Brittain SM, Jones MD, Ou L (2019). Harnessing the anti-cancer natural product nimbolide for targeted protein degradation. Nature chemical biology.

[15] Li P, Zhen Y, Kim C, Liu Z, Hao J, Deng H (2023). Nimbolide targets RNF114 to induce the trapping of PARP1 and synthetic lethality in BRCA-mutated cancer. Science advances.

[16] Luo M, Spradlin JN, Boike L, Tong B, Brittain SM, McKenna JM (2021). Chemoproteomics-enabled discovery of covalent RNF114-based degraders that mimic natural product function. Cell Chem Biol.

[17] Camp RL, Dolled-Filhart M, Rimm DL (2004). X-tile: a new bio-informatics tool for biomarker assessment and outcome-based cut-point optimization. Clinical cancer research: an official journal of the American Association for Cancer Research.

[18] Rossow KL, Janknecht R (2001). The Ewing's sarcoma gene product functions as a transcriptional activator. Cancer research.

[19] Hsu JL, Hung MC (2016). The role of HER2, EGFR, and other receptor tyrosine kinases in breast cancer. Cancer metastasis reviews.

[20] Swain SM, Shastry M, Hamilton E (2023). Targeting HER2-positive breast cancer: advances and future directions. Nature reviews Drug discovery.

[21] Ferrando-Díez A, Felip E, Pous A, Bergamino Sirven M, Margelí M (2022). Targeted Therapeutic Options and Future Perspectives for HER2-Positive Breast Cancer. Cancers (Basel).

[22] Blackwell KL, Dewhirst MW, Liotcheva V, Snyder S, Broadwater G, Bentley R (2004). HER-2 gene amplification correlates with higher levels of angiogenesis and lower levels of hypoxia in primary breast tumors. Clinical cancer research: an official journal of the American Association for Cancer Research.

[23] Alameddine RS, Otrock ZK, Awada A, Shamseddine A (2013). Crosstalk between HER2 signaling and angiogenesis in breast cancer: molecular basis, clinical applications and challenges. Current opinion in oncology.

[24] Nasir A, Holzer TR, Chen M, Man MZ, Schade AE (2017). Differential expression of VEGFR2 protein in HER2 positive primary human breast cancer: potential relevance to anti-angiogenic therapies. Cancer Cell Int.

[25] Fertig EJ, Lee E, Pandey NB, Popel AS (2015). Analysis of gene expression of secreted factors associated with breast cancer metastases in breast cancer subtypes. Sci Rep.

[26] Rugo HS, Chien AJ, Franco SX, Stopeck AT, Glencer A, Lahiri S (2012). A phase II study of lapatinib and bevacizumab as treatment for HER2-overexpressing metastatic breast cancer. Breast cancer research and treatment.

[27] He L, Shen X, Liu Y, Gao L, Wu J, Yu C (2022). The reversal of anti-HER2 resistance in advanced HER2-positive breast cancer using apatinib: two cases reports and literature review. Translational cancer research.

[28] Kodack DP, Chung E, Yamashita H, Incio J, Duyverman AM, Song Y (2012). Combined targeting of HER2 and VEGFR2 for effective treatment of HER2-amplified breast cancer brain metastases. Proc Natl Acad Sci U S A.

[29] Kim Y, Kang YS, Lee NY, Kim KY, Hwang YJ, Kim HW (2015). Uvrag targeting by Mir125a and Mir351 modulates autophagy associated with Ewsr1 deficiency. Autophagy.

[30] Lee J, Nguyen PT, Shim HS, Hyeon SJ, Im H, Choi MH (2019). EWSR1, a multifunctional protein, regulates cellular function and aging via genetic and epigenetic pathways. Biochimica et biophysica acta Molecular basis of disease.

[31] Filion C, Motoi T, Olshen AB, Laé M, Emnett RJ, Gutmann DH (2009). The EWSR1/NR4A3 fusion protein of extraskeletal myxoid chondrosarcoma activates the PPARG nuclear receptor gene. The Journal of pathology.

[32] Henon C, Vibert J, Eychenne T, Gruel N, Colmet-Daage L, Ngo C (2024). Single-cell multiomics profiling reveals heterogeneous transcriptional programs and microenvironment in DSRCTs. Cell reports Medicine.

[33] Lee SB, Kolquist KA, Nichols K, Englert C, Maheswaran S, Ladanyi M (1997). The EWS-WT1 translocation product induces PDGFA in desmoplastic small round-cell tumour. Nature genetics.

[34] Matsui Y, Ueda T, Kubo T, Hasegawa T, Tomita Y, Okamoto M (2006). A novel type of EWS-CHOP fusion gene in myxoid liposarcoma. Biochemical and biophysical research communications.

[35] Warmke LM, Strike SA, Fayad LM, Ahlawat S, Liu YJ, Mata DA (2024). Undifferentiated Round Cell Sarcoma With CRTC1::SS18 Fusion: Expanding Clinicopathologic Features of a Rare Translocation Sarcoma With Prominent Desmoplastic Stroma. Modern pathology: an official journal of the United States and Canadian Academy of Pathology, Inc.

