# Supplementary material for: RNF114 Interacts with EWSR1 to Regulate VEGFR2 in HER2-positive Breast Cancer
Source: J Cancer. 2025 Feb 28;16(6):1888–904. doi: 10.7150/jca.106001 (PMC11905403; doi:10.7150/jca.106001)
Supplement: Supplementary file 1 — Supplementary methods, figures and tables. [file jcav16p1888s1.pdf]

Supplementary Materials

Doc S1

Western blot analysis

Cellular protein was extracted using universal protein extraction buffers (Bioteke, China) containing protease inhibitor cocktail (Roche, Switzerland). The protein lysates were separated on 5-12.5% SDS-PAGE gels and transferred onto a polyvinylidene diffuoride (PVDF) membrane (Millipore, USA). The transferred membranes were incubated for 1 h in 5% non-fat milk and then sequentially incubated with specific primary antibodies followed by horseradish peroxidase (HRP)-conjugated secondary antibodies.

RT–PCR and real-time quantitative RT–PCR

Total RNA was extracted using Nucleic Acid Extraction Kit (BioTeke, China) and the concentration was detected with NanoDrop^{TM} 2000 Spectrophotometer (Thermo, USA). Reverse transcription and real-time PCR were performed using Hifair$^{@}$ III 1st Strand cDNA Synthesis Super Mix (YEASEN, China) and Hieff UNICON$^{@}$ Universal Blue qPCR SYBR Green Master Mix (YEASEN, China). The transcript levels were normalized using the ΔΔCt method.

Cell assays for proliferation, migration and invasion

For CCK-8 assay, cell viability following various treatments was assessed using the Cell Counting Kit (BIOGROUND, China). Cells were first resuspended for counting and then seeded into 96-well plates at a density of 3000 cells per well. After incubation for 24, 48 and 72 hours in a humidified chamber with 5% CO_2 at 37°C, 10 μl of assay reagent was added to each well. The plates were then incubated in the dark for 90 minutes at 37°C. The absorbance (optical density) of each well was then measured at 450 nm using a microplate reader. For cell migration assay, cells were cultured in 12-well plates, and a "wound" was created in the cell monolayer. After adding fresh medium, the cells were imaged using an inverted microscope (Olympus, Japan) at 24, 48, and 72h. For cell invasion assay, 5000 serum-free media cells were added to the upper chamber after the Matrigel (ABW, China) solidified, while 20% FBS media was added to the lower chamber. Cells were incubated at 37°C with 5% CO_2 for 48h. After fixing with 70% ethanol and staining with crystal violet, images were captured using a fluorescence inverted microscope (Olympus, Japan).

Co-immunoprecipitation

Cells were lysed in universal protein extraction buffers (BioTeke, China) containing protease inhibitor cocktail (Roche, Switzerland). Protein concentration was detected using a BCA Protein Assay Kit (Beyotime Biotechnology, China). Extracted proteins were immunoprecipitated with a special primary antibody and protein A+G agarose beads (Beyotime, China). The bead-bound proteins were then released and analyzed by Western blot.

RNA-seq assay

Total RNA was extracted from (1×10^6) tumor cells of stable RNF114 knockdown and control SKBR3 cells using RNA Extraction Kit (BioTeke, China). Library preparation and transcriptome sequencing were performed on an Illumina HiSeq4000 platform at SeqHealth (Wuhan, China).

Transmission electron microscopy (TEM)

TEM was employed to observe the ultrastructural changes in stable RNF114 knockdown SKBR3 and BT474 cells. Cells were collected and washed three times with precooled PBS, and then fixed in 2.5% glutaraldehyde for 24 hours. The samples were subsequently sent to Chengdu Li Lai Technology (Chengdu, China) for transmission electron imaging.

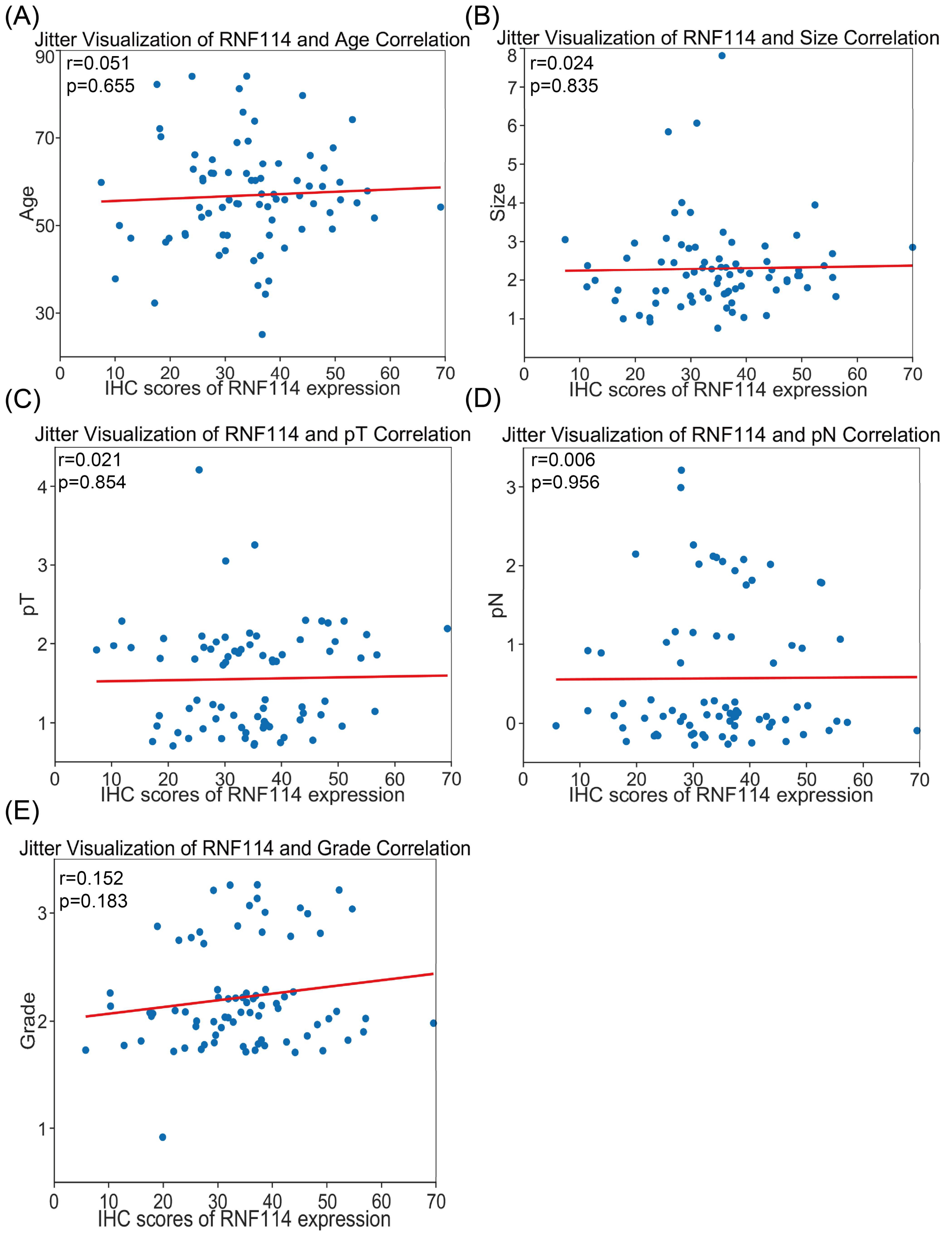

Figure S1 Correlation between RNF114 IHC expression scores and clinical parameters. Age (A), Size (B), pT (C), pN (D), Grade (E). The scatter plot shows individual data points with a jittered distribution to enhance visibility.

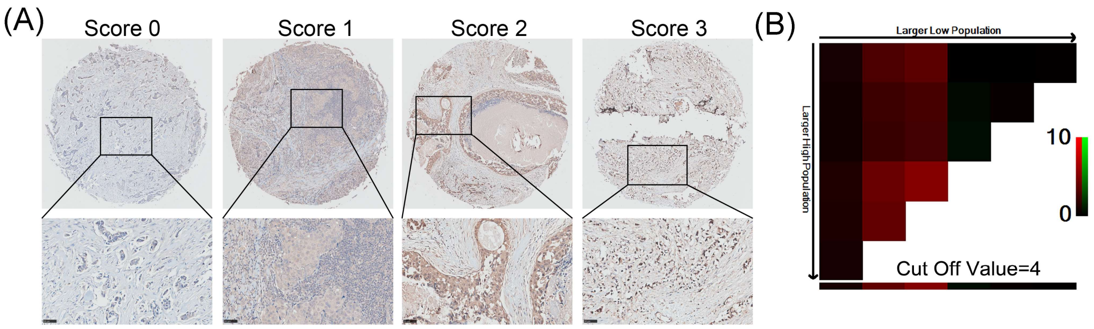

Figure S2 IHC scores of breast cancer tissue microarray were analyzed using X-tile software. (A) RNF114 expression levels in 78 breast cancer patients were scored by staining intensity (0-3) and area (0-4) of the staining with tissue microarray technology and immunohistochemistry. Representations of intensity quantification: 0 (no color reaction), 1 (mild reaction), 2 (moderate reaction), or 3 (intense reaction). (B)The final scores of all samples were analyzed using X-tile software. It is showed that the maximum chi-square value is obtained when the score is 4. The scores of all samples were successively divided into the low-expression RNF114 group (score 0-4) and the high-expression RNF114 group (score 4-12).

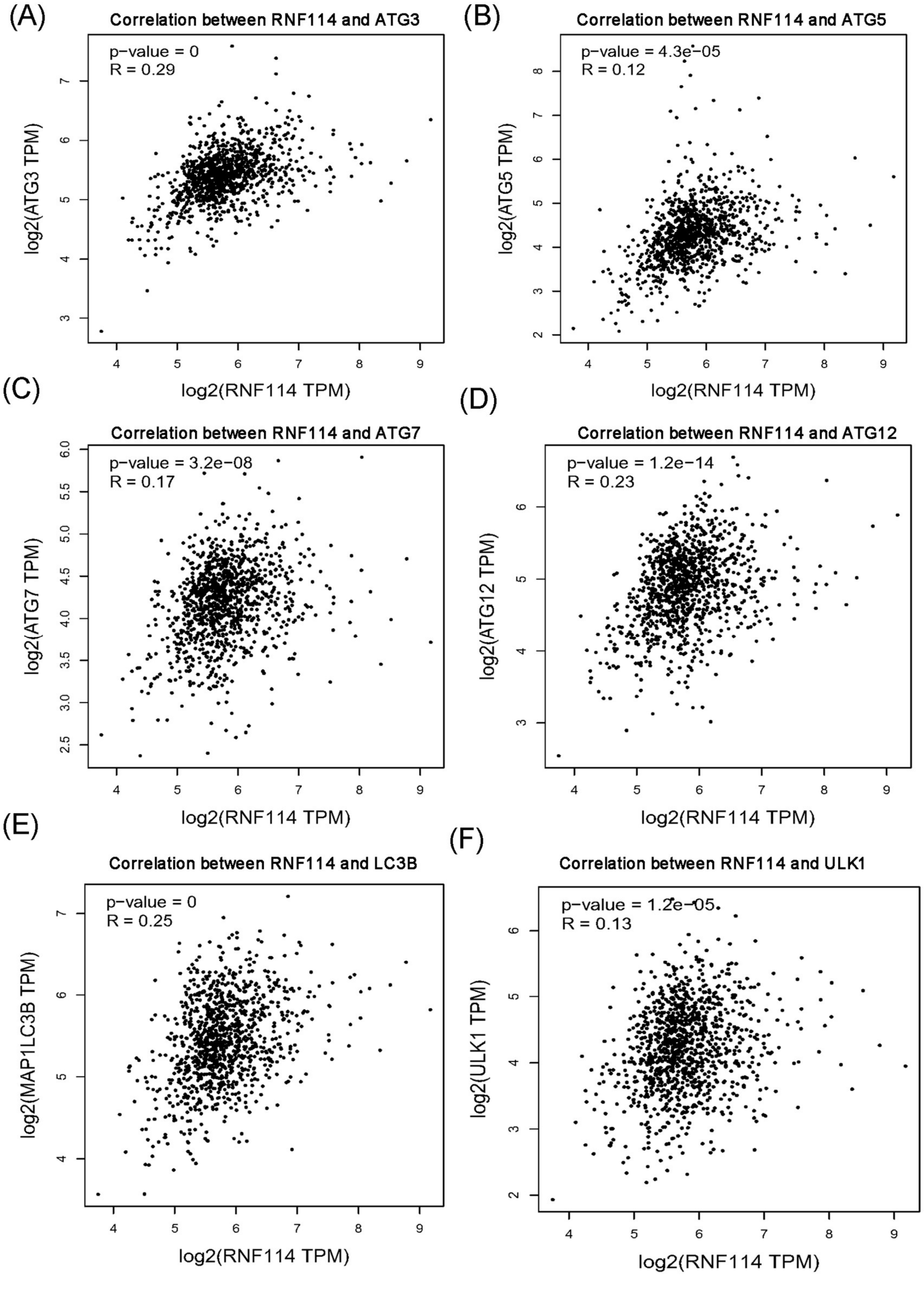

Figure S3 Correlation analysis of *RNF114* expression with several autophagy-related genes. *ATG3*(A), *ATG5*(B), *ATG7*(C), *ATG12*(D), *LC3B*(E) and *ULK1* (F).

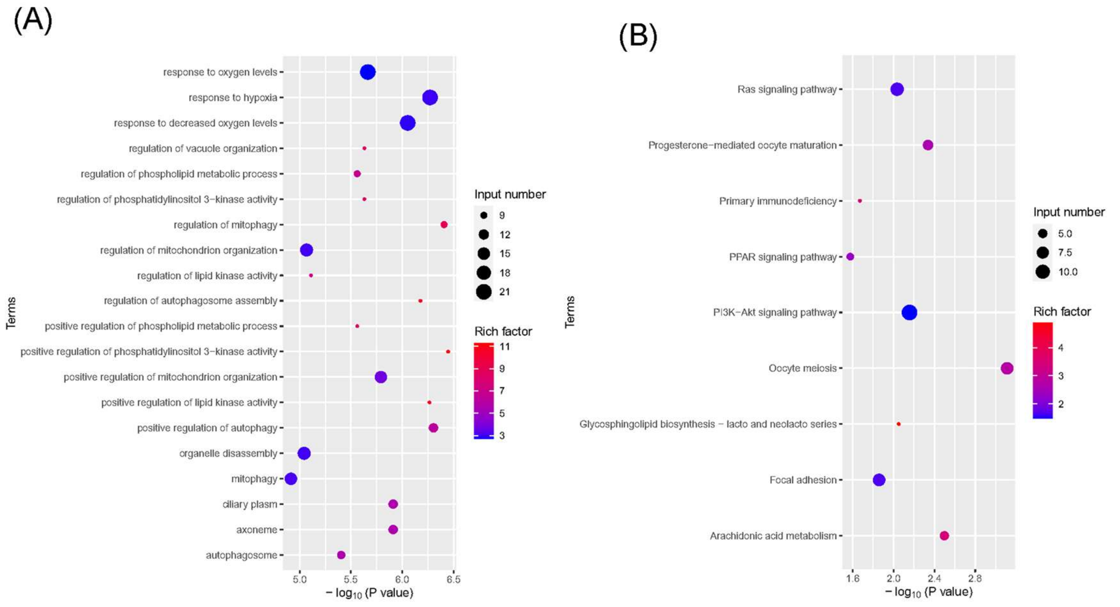

Figure S4 GO and KEGG analysis of significantly downregulated genes in RNF114 stable knockdown SKBR3 cells. (A) Gene ontology (GO) enrichment analysis. (B) Kyoto Encyclopedia of Genes and Genomes (KEGG) enrichment analysis

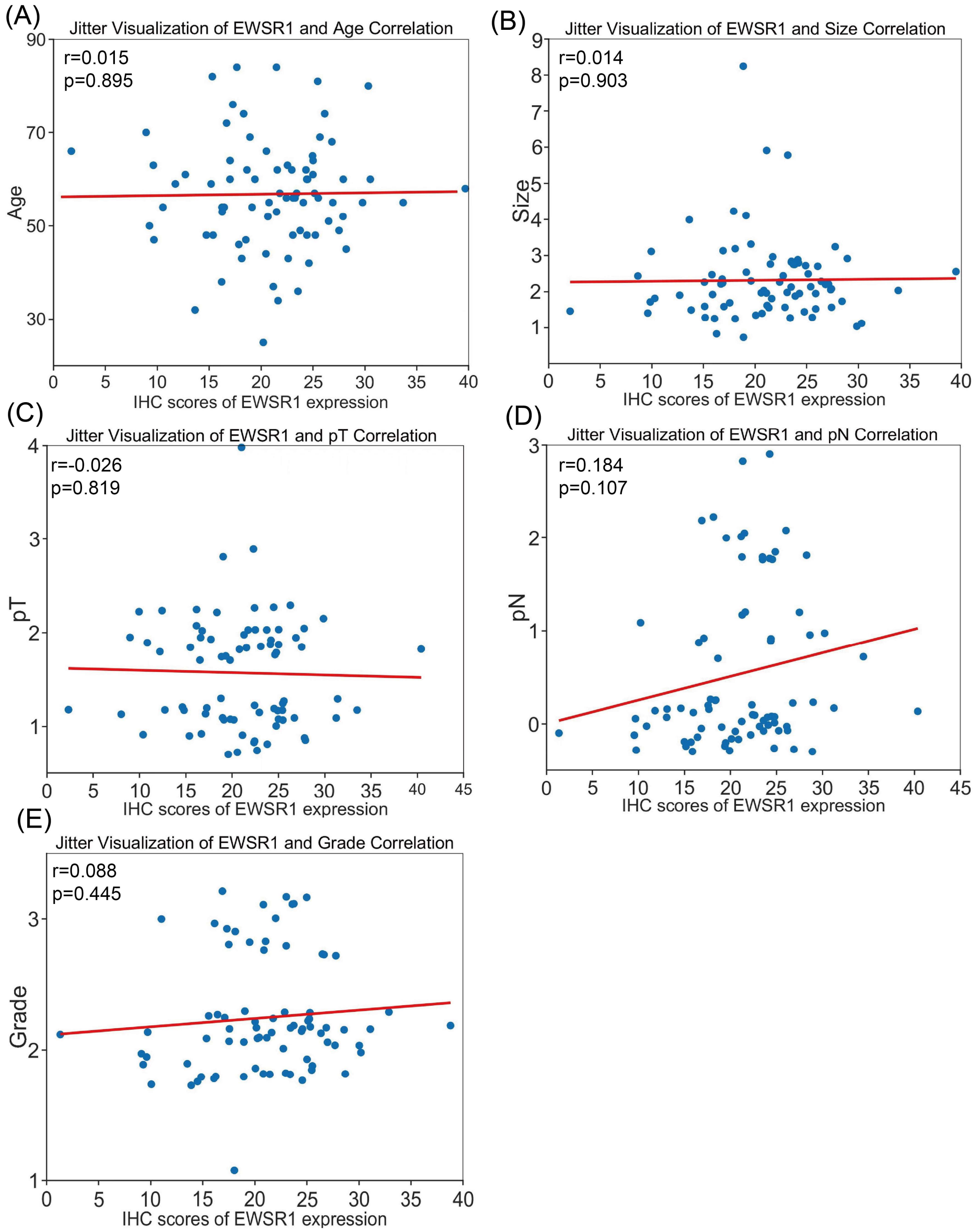

Figure S5 Correlation between EWSR1 IHC expression scores and clinical parameters. Age (A), Size (B), pT (C), pN (D), Grade (E). The scatter plot shows individual data points with a jittered distribution to enhance visibility.

Supplementary Table 1 Clinical pathological indexes of the 78 breast cancer patients

Characteristic		N (%)
Age		
	<=60	52(67)
	>60	26(33)
Size (cm)		
	<=3	77(99.7)
	>3	1(1.3)
pT		
	T1	38(48.7)
	T2	37(47.4)
	T3	2(2.6)
	T4	1(1.3)
pN		
	N0	52(66.7)
	N1	11(14.1)
	N2	12(15.4)
	N3	3(3.8)
Grade		
	1	1(1.3)
	2	58(74.4)
	3	19(24.3)
TNM-stage		
	I	25(32)
	II	31(40)
	III	15(19)
	IV	7(9)

Supplementary Table 2 The primary antibodies used in this study

Antibodies	Source	Identifier
**RNF114**	Santa Cruz Biotechnology	sc-101116
**EWSR1**	ZEN BIO	R24256
**VEGFR2**	HUABIO	HO0118
**P-VEGFR2**	Cell Signaling Technology	2478s
**ERK1/2**	ZEN BIO	343830
**P-ERK1/2**	ZEN BIO	310065
**MEK1/2**	ZEN BIO	384949
**P-MEK1/2**	ZEN BIO	310050
**m-TOR**	Proteintech	66888-1-Ig
**P-mTOR**	Cell Signaling Technology	5536s
**ULK1**	Cell Signaling Technology	8054s
**P-ULK1**	Cell Signaling Technology	5869s
**LC3B**	ZEN BIO	382687
**Flag**	ZEN BIO	R24091
**EGFP**	ZEN BIO	300943
**GAPDH**	ZEN BIO	R24402
**Tubulin**	ZEN BIO	250009

Supplementary Table 3 The primers used in qPCR

Gene	Primers	Nucleotide sequence 5'-3'
RNF114	Forward	GTGTCCCGTGTGCTTAGAGG
	Reverse	CTCTGTGCTCTCGATCTGCC
VEGFR2	Forward	GTGATCGGAAATGACACTGGAG
	Reverse	CATGTTGGTCACTAACAGAAGCA
GAPDH	Forward	ACGGATTTGGTCGTATTGGG
	Reverse	CGCTCCTGGAAGATGGTGAT

Supplementary Table 4 The primers used in ChIP-qPCR

Primers	Nucleotide sequence 5'-3'
***VEGFR2* ChIP F1**	ACAGAAAACCAGAAGGAACGA
***VEGFR2* ChIP R1**	GTGTGTGGACTTCTTGTGGCA
***VEGFR2* ChIP F2**	AGAAGGAACGAATGTGGTCAG
***VEGFR2* ChIP R2**	GTGTGTGGACTTCTTGTGGCA
***VEGFR2* ChIP F3**	AAGGAACGAATGTGGTCAGGA
***VEGFR2* ChIP R3**	GTGTGTGGACTTCTTGTGGCA

Supplementary Table 5 EWSR1 regulation of VEGFR2 transcription with 24 potential binding sites predicted using Animal TFDB database.

hTFtarget	Start	End	Strand	P Value	Predicted sequence
**VEGFR2**	256	274	+	4.19E-07	GAAAAGATGGAAGGATGGA
**VEGFR2**	1068	1092	+	7.02E-07	AGGAACGAATGTGGTCAGGAAGGAA
**VEGFR2**	152	167	+	1.06E-05	AGCAGGAAGAGAGGAG
**VEGFR2**	1070	1089	+	1.61E-05	GAACGAATGTGGTCAGGAAG
**VEGFR2**	1066	1085	+	1.68E-05	GAAGGAACGAATGTGGTCAG
**VEGFR2**	1216	1231	-	1.72E-05	GGGGAAGGAGGCCAGC
**VEGFR2**	257	276	+	1.77E-05	AAAAGATGGAAGGATGGAGC
**VEGFR2**	2216	2234	-	2.01E-05	AGAAGGAGGCGCGGAGGTG
**VEGFR2**	260	275	+	2.27E-05	AGATGGAAGGATGGAG
**VEGFR2**	258	273	+	2.39E-05	AAAGATGGAAGGATGG
**VEGFR2**	1074	1093	+	4.01E-05	GAATGTGGTCAGGAAGGAAT
**VEGFR2**	1217	1228	+	4.24E-05	CTGGCCTCCTTC
**VEGFR2**	1923	1938	-	4.44E-05	GGAGCGGAGGAGGGGC
**VEGFR2**	1072	1096	+	5.57E-05	ACGAATGTGGTCAGGAAGGAATTGG
**VEGFR2**	260	278	+	5.63E-05	AGATGGAAGGATGGAGCTT
**VEGFR2**	471	486	+	5.84E-05	GAGGCAGGAGGATGGA
**VEGFR2**	256	271	+	6.09E-05	GAAAAGATGGAAGGAT
**VEGFR2**	1924	1942	-	6.54E-05	GGCCGGAGCGGAGGAGGGG
**VEGFR2**	1052	1076	+	6.57E-05	TGGACAGAAAACCAGAAGGAACGAA
**VEGFR2**	1062	1081	+	6.97E-05	ACCAGAAGGAACGAATGTGG
**VEGFR2**	152	170	+	7.52E-05	AGCAGGAAGAGAGGAGTTT
**VEGFR2**	1077	1092	+	7.72E-05	TGTGGTCAGGAAGGAA
**VEGFR2**	150	165	+	8.39E-05	CTAGCAGGAAGAGAGG
**VEGFR2**	2221	2236	-	8.81E-05	AGAGAAGGAGGCGCGG
